# *De Novo* Design of Potent, Insecticidal Synthetic Mimics of the Spinosyn Macrolide Natural Products

**DOI:** 10.1038/s41598-018-22894-6

**Published:** 2018-03-20

**Authors:** Gary D. Crouse, David A. Demeter, Geno Samaritoni, Casandra L. McLeod, Thomas C. Sparks

**Affiliations:** 1 Dow AgroSciences, Discovery Research, 9330 Zionsville Road, Indianapolis, IN 46268 USA; 2 Present Address: 5069 E 146th St Noblesville IN 46062, Indianapolis, IN USA; 3 Present Address: Department of Chemistry and Chemical Biology, Indiana University - Purdue University Indianapolis, 402 N. Blackford Street, Indianapolis, IN 46202 USA; 4 Present Address: 6034 Haverford Ave, Indianapolis, IN 46220 USA

## Abstract

New insect pest control agents are needed to meet the demands to feed an expanding global population, to address the desire for more environmentally-friendly insecticide tools, and to fill the loss of control options in some crop-pest complexes due to development of insecticide resistance. The spinosyns are a highly effective class of naturally occurring, fermentation derived insecticides, possessing a very favorable environmental profile. Chemically, the spinosyns are composed of a large complex macrolide tetracycle coupled to two sugars. As a means to further exploit this novel class of natural product-based insecticides, molecular modeling studies coupled with bioactivity-directed chemical modifications were used to define a less complex, synthetically accessible replacement for the spinosyn tetracycle. These studies lead to the discovery of highly insecticidal analogs, possessing a simple tri-aryl ring system as a replacement for the complex macrolide tetracycle.

## Introduction

Herein we demonstrate for the first time that the fermentation-derived, complex macrocyclic lactone tetracycle core of the spinosyn class of natural product (NP) insecticides^1–3^ can be mimicked by a simple, synthetic tri-aryl ring system. Further, the insecticidal activity of these new non-macrolide synthetic spinosyns exceeds that of the commercialized NP spinosyn insecticide, spinosad. These new spinosyn-mimics are the first examples of simplifying a large macrolide in the agrochemical arena, and as far as we are aware, also in the pharmaceutical arena of macrolide antibiotics. Importantly, our results suggest that it may also be possible to simplify other macrolide-based compounds including macrolide insecticides and antibiotics eliminating the need for fermentation-based starting materials. Thus, we demonstrate that new options are available for large macrolide NPs providing an opportunity for new molecules that maintain the mode of action of the NP, but possess attributes beyond those of the NP, including improved physical properties, efficacy, bioavailability, and spectrum.

**Figure 1:**
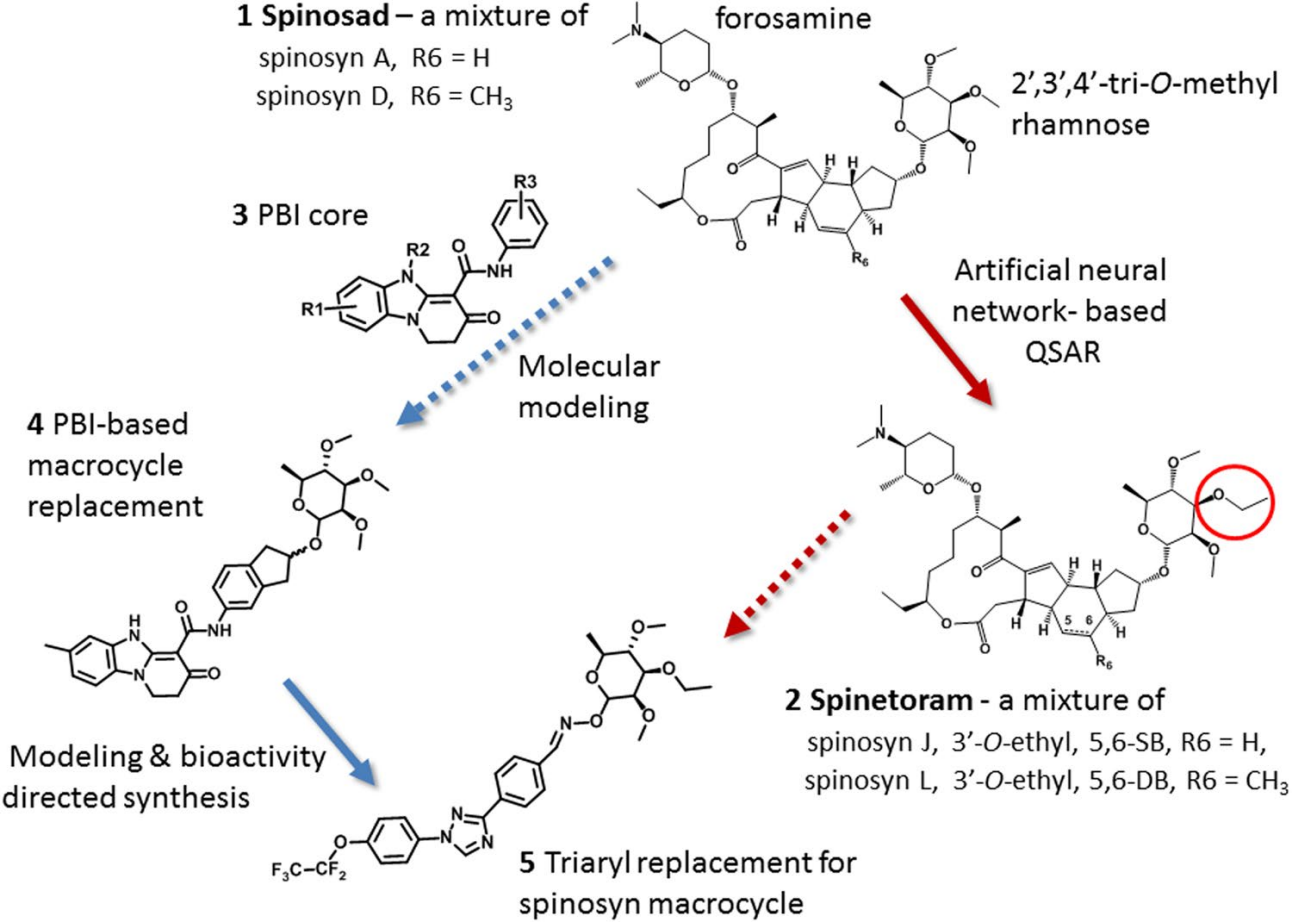
Structures of commercial insecticides, spinosad (**1**) and spinetoram (**2**) and the conceptual route to the discovery of the spinosyn mimics. Spinosad (**1**) is a natural product from fermentation composed of a macrolide tetracycle and two sugars; forosamine and 2′, 3′, 4′-tri-*O*-methyl rhamnose. Spinetoram (**2**) is a semi-synthetic modification of a fermentation derived mixture of spinosyns. As a substitution for the natural macrolide core of the spinosyns, the initial PBI core (**3**) was coupled to an indane linked with the 2′, 3′, 4′-tri-*O*-methyl rhamnose for form a PBI-based spinosyn mimic (**4**); further evolution of the chemistry lead to the simplified tri-aryl core replacement of macrolide tetracycle incorporating an oxime linker to the 2′, 3′, 4′-tri-*O*-methyl rhamnose (**5**).

The spinosyns (e.g. spinosad **1**; Fig. 1) are a novel class of insecticidal macrolide natural products that are highly effective against a wide range of pest insects and possess very favorable toxicological and environmental profiles^1–3^. Synthetic modification of the spinosyn structure, driven by artificial neural network-based quantitative structure activity relationships (QSAR), led to the discovery of spinetoram **2** (Fig. 1), a more potent, broader spectrum semi-synthetic derivative^3,4^. Although semi-synthetic modifications, genetic manipulations and additional natural product discovery efforts have resulted in numerous other spinosyn derivatives, all retain the macrolide-based tetracycle core^5,6^. Further commercial exploitation of this favorable mode of action is limited due to the inherent costs, accessibility and physical properties of the fermentation-derived macrolides. Herein we describe a successful computationally aided approach to the discovery of synthetic analogs of the spinosyns.

## Design - Synthetic Spinosyn Mimics

The initial concept for the synthetic spinosyns was based on the idea that a simple, rigid scaffold mimicking only part of the macrocycle might provide a framework to replace the entire spinosyn macrolide tetracycle, leading to far simpler molecules. Initial modeling suggested that the pyridobenzimidazole (PBI **3**, Fig. 1) core might be suitable as a potential replacement for the spinosyn macrolide tetracycle because they share two key hydrogen bond acceptors that overlap based on a published λ-aminobutyric acid (GABA_A_) benzodiazepine site pharmacophore model^7^. Additionally, PBIs were known to interact at the benzodiazepine site in vertebrate GABA_A_ receptors^7,8^. While the spinosyns appear to act primarily through the insect nicotinic acetylcholine receptors (nAChR)^5,9^, there is also evidence that they can interact with some insect GABA receptors^10^. Both nAChRs and GABA receptors belong to the ‘Cys-loop’ family of pentameric ligand-gated ion channels (LGICs) that also include receptors for 5-hydroxytryptamine (5-HT),and glycine^11^.

**Figure 2:**
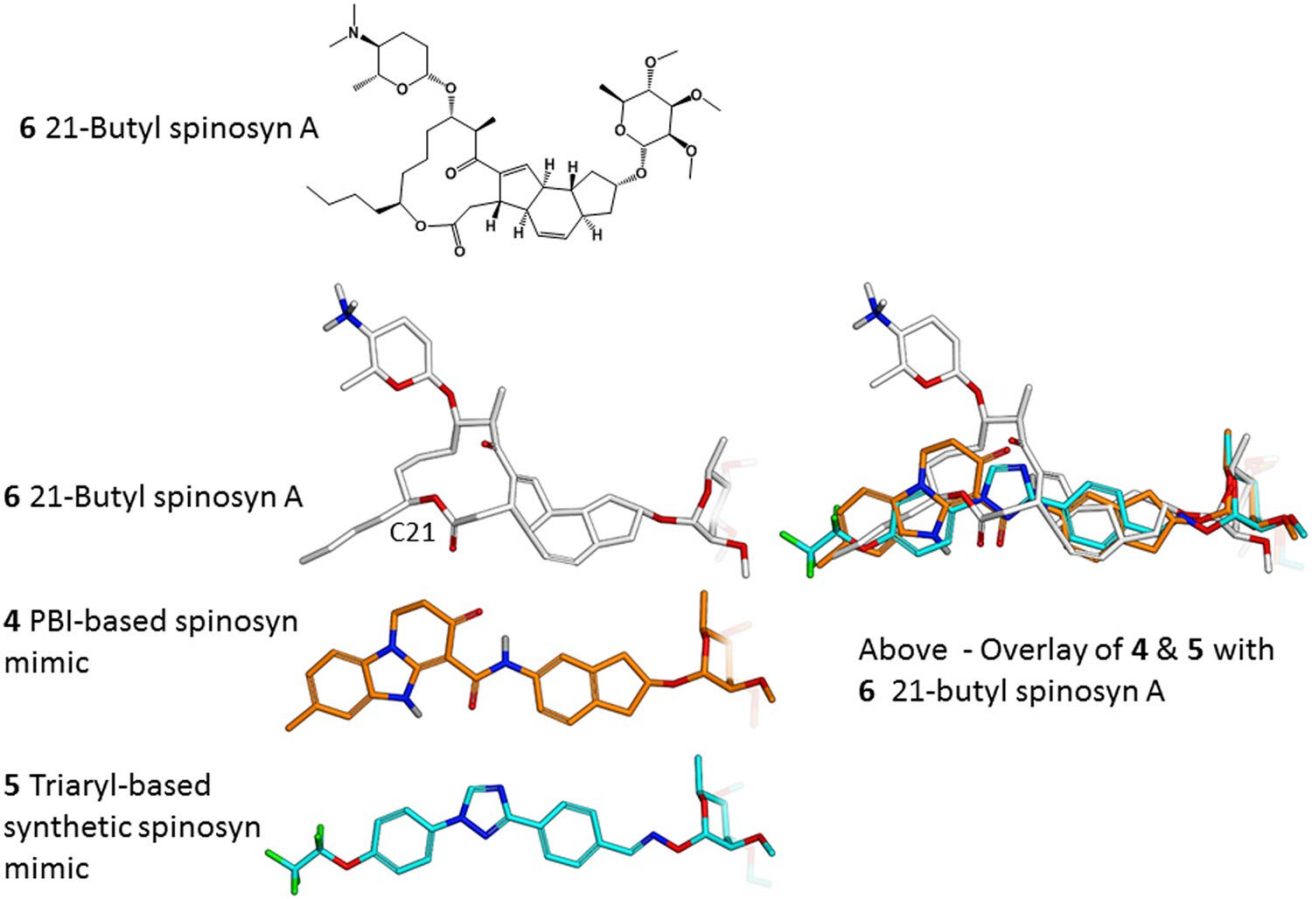
3D-structures and overlay of 21-butyl spinosyn A (**6**), the initial PBI-based spinosyn scaffold (**4)** and the synthetic spinosyn mimic (**5)** possessing a triaryl replacement for the spinosyn macrocycle. All of the structures **4**, **5**, **6** are aligned to the 2′, 3′, 4′-tri-*O*-methyl rhamnose.

Molecular modeling overlays of the basic PBI core **3** (Figs 1 and 2) showed that placement of the rhamnose in its putative binding pocket could be accomplished through the addition of a 2-indanyl linker between the PBI core **3** and the rhamnose sugar of spinosyn A resulting in the PBI-based spinosyn mimic **4**. Although **4** was insecticidally inactive *in vivo* when initially bioassayed (larval diet bioassay at a screening dose of 12.5 µg/cm^2^) (Table 1), it did display the correct symptoms of poisoning consistent with spinosad (fine body tremors, rapid movement of the mouth parts, and paralysis) when injected into larvae of *Spodoptera exigua* (beet armyworm). The correct symptomology in the injection bioassay provided the impetus for continued exploration of this motif for a synthetic spinosyn mimic. Further elaboration on the structure of **4** led to a series of successive analogs that, through repeated computational analysis and bioactivity-directed synthetic refinement (injection assays and *in vivo* diet assays), evolved into the simplified tri-aryl motif exemplified by the triaryl-based spinosyn mimic **5** (Figs 1 and 2). As shown in Fig. 2, both **4** and **5** can overlay with the spinosyn macrolide tetracycle. Interestingly, **4** and **5** overlays do not extend into the space occupied by the forosamine sugar. Rather the overlays suggest that these spinosyn mimic scaffolds fit best into a space defined by substituents at the C21 position of the macrocycle. Studies with the C21-butyl **6**/butenyl spinosyns^12^ and range of other analogs possessing bulky substituents at the C21 position^6,13,14^ suggest that there is a sizable pocket around the C21 position in the spinosyn binding site.

**Table 1:** Insecticidal activity of spinosyns and synthetic mimics to larvae of *S. exigua* (*Se*)*, H. zea* (*Hz*), and adults of two strains (WT and spinosad-resistant) of *D. melanogaster* (*Dm*).

Compound	LC_50_ µg/cm^2^ (95% FL)^1^	LC_50_ µg/cm^2^ (95% FL)	LC_50_ ppm (95% FL)	LC_50_ ppm (95% FL)	RR^4^
	*Se*	*Hz*	WT *Dm*^2^	SR-*Dm*^3^	
**1** spinosad	0.052	0.058	0.035	10.9	311
	(0.021–0.083) n = 432	(0.045–0.075) n = 320	(0.0091–0.123) n = 793	(9.29–13.4) n = 744	
**2** spinetoram	0.0077	0.0087	0.025	3.52	139
	(0.0044–0.015) n = 688	(0.0073–0.010) n = 672	(0.0204–0.032) n = 562	(2.79–4.38) n = 541	
**4**	>12.5 n = 32	>12.5 n = 32	—	—	—
**5**	0.0046	0.0034	0.0048	0.55	114
	(0.0038–0.0055) n = 400	(0.0029–0.0040) n = 400	(0.0035–0.0057) n = 325	(0.39–0.72) n = 318	

^1^LC_50_s were calculated using probit analysis^24^. FL = fiducial limits, n = number of insects tested.

^2^Wild type (susceptible) adult *D. melanogaster*.

^3^Spinosad-resistant adult *D. melanogaster* – see ref.^9^ for details.

^4^Resistance ratio: LC_50_ SR-*Dm*/LC_50_ WT *Dm*.

## Biological Characterization

Compared to the natural product spinosad **1**, the spinosyn mimic **5** is an order of magnitude more active. Further, the spinosyn mimic **5** also exhibits insecticidal activity comparable to or slightly better than the more insecticidally active semi-synthetic spinosyn-based product, spinetoram **2** (Table 1). The excellent insecticidal efficacy of the synthetic spinosyn mimic **5** suggests that there is little penalty for omitting the forosamine sugar or a forosamine bioisostere. The same cannot be said for the naturally occurring spinosyns in that removal of the forosamine sugar results in a very large loss in insecticidal activity^2,5^.

The spinosyns act at an allosteric site on the α6 subunit of the insect nAChR^5,9,15^ which is a mode of action that has remained unique among agrochemicals for 20 years^16^. Thus, if the synthetic spinosyn mimics like **5** are indeed functioning as synthetic spinosyns, insects with an altered spinosyn target site should exhibit resistance to **5** as it does with spinosad **1** and spinetoram **2**. As noted in Table 1, a strain of *Drosophila melanogaster* that possesses a resistance inducing target site mutation (nAChR, Dα6)^9^, also exhibits the same high degree of cross-resistance to the spinosyn mimic **5** as it does to the spinosyns spinosad **1** and spinetoram **2** (Table 1). These data strongly indicate that the simplified molecule **5** has retained the same mode of action as the natural spinosyns.

Interestingly, when the *in vivo* insecticidal activity of the initial synthetic model mimic **4** (LC_50_ > 12.5 µg/cm^2^) is compared to that of the spinosyn synthetic mimic **5** (LC_50_ = 0.0046 & 0.0034 μg/cm^2^ for larvae of *S. exigua* and *H. zea*, respectively), there has been a >2700-fold improvement in insecticidal activity against these key pest insect larvae (Table 1). Likewise, this synthetic spinosyn mimic **5** is also >10-fold more insecticidal than the natural product (spinosad **1**), and is comparable to the more efficacious semi-synthetic commercial derivative, spinetoram **2** (Table 1). Thus, the efficacy achieved with this synthetic spinosyn scaffold **5** clearly demonstrates that it is possible to replace the complex macrocycle of the spinosyn NP with a chemically simpler structure.

## Conclusion

In conclusion we have shown that the complex macrocyclic lactone tetracycle core of the spinosyns can be mimicked by a synthetically simpler tri-aryl scaffold yielding new molecules that maintain the mode of action of the spinosyns, but exceed the insecticidal activity of the commercial NP, spinosad. We are not aware of any other successful macrolide mimics in the agricultural arena, nor in the macrolide antibiotic arena. Implicit in our results is the concept that it may be possible to simplify other macrolide-based compounds such as macrolide antibiotics (e.g. erythromycin), offering an alternative to such NP-based medicines, and providing an opportunity to alter a wide range of properties, including physical attributes, efficacy, bioavailability, and spectrum.

## Materials and Methods

### Molecular Modeling

The X-ray structure of spinosyn A (**1)** was minimized using the MMFF94 force field^17–21^ in Sybyl-X 2.1.1^22^ (minimization-method = Powell, min-energy-change = 0.00001, dielectric = 2.0*R, maximum-iterations = 1000000). Default values were used for all other variable parameters. The ethyl at the 21 position was converted to butyl. Leaving the 21-butyl substituent extended, the three non-eclipsed low energy conformers were built and minimized as above. The unsubstituted PBI-motif analog was built and minimized in the same manner. It was RMS fit manually to the 21-butyl-spinosyn A (**6)** conformers using the 2 carbonyl oxygens in each structure and the centroids of the amide phenyl in the PBI (**3**) and indanyl 6-membered ring and the overlay with the best volume overlap was selected. Adding a fused five-membered ring to the PBI phenyl group forming an indane linker between the rhamnose and PBI core yielded the synthetic mimic (**4)**. **4** was minimized and RMS fit to 21-butyl-spinosyn A (**6)** using the 2 carbonyl oxygens in each structure, the rhamnose oxygens and the terminal methyl group on the PBI core and the butyl side chain. **5** was minimized and RMS fit to PBI-SM using the 2 central ring nitrogens with the 2 carbonyl oxygens, the terminal fluorine atom with the terminal methyl carbon and the rhamnose oxygens.

### Bioassays

Injection Assay - Fourth instar larvae of *Spodoptera exigua* (beet armyworm) were injected, using a 10 µL Hamilton 1701SN, 33/0.5′′ syringe, with 0.5 µL of the test compound in dimethyl sulfoxide (DMSO) (10 µg/larva). For each compound six larvae were injected along the side of the abdomen, and then held individually in a clear, six-well microtiter plate with a small piece of artificial diet and covered with a plastic lid (22.2 °C, and a 14:10 Light:Dark photoperiod). Larvae injected with 0.5 µL of DMSO were as controls for solvent effects. Following injection, larvae were examined under a dissecting microscope at 1, 3, 6, 24 and 48 hours for symptoms and mortality. Treatments were replicated two or three times and the results were averaged.

The diet bioassays were run as described previously^23^ using second instar larvae of *S. exigua* and *Heliothis zea* (corn earworm), with 16 larvae per dose, 4–5 doses, replicated two to three times. The *Drosophila* bioassays were run using adult *D. melanogaster* as described previously^9^ with 10–16 flies per dose and 4–5 doses replaced on four separate occasions. LC_50_s and the associated 95% fiducial limits were calculated using probit analysis^24^.

#### Compounds

Spinosad (**1)** and spinetoram (**2)** were from Dow AgroSciences. The synthesis of the triaryl-based spinosyn mimic (**5**) is described by Crouse *et al*.^25,26^. The synthesis of PBI (**3**) was as described in Scott *et al*.^7^. The PBI-based spinosyn mimic (**4**) was prepared as described below.

2′, 3′, 4′-Tri-*O*-methyl-L-rhamnopyranoside was obtained by hydrolysis of spinosad under previously described conditions^23^. All reactions were executed under N_2_ in dried glassware. Nuclear magnetic resonance spectra were recorded on a Varian Gemini 300 spectrometer unless otherwise noted. Chemical shifts were reported in ppm downfield from an internal tetramethylsilane. Mass spectra were obtained using a Hewlett Packard 1100 MSD liquid chromatograph/mass spectrometer.

3′, 4′, 5′-trimethoxy-2-methyl-6-(5-nitro-indan-2-yloxy)-tetrahydro-pyran.
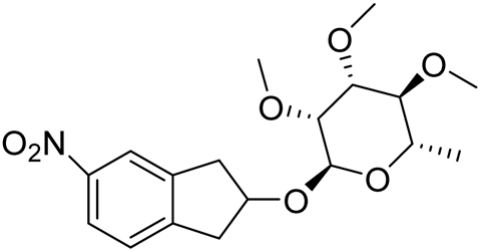


The 5-nitro-2-indanol (3.6 g, 0.02 mol, 1 eq.) and 1-(t-butyldimethyl-silyloxy) rhamnose (8.0 g, 25 mmol, 1.25 eq.) were dissolved in dichloromethane (DCM) (100 mL) and trimethylsilyl trifluoromethanesulfonate (1.3 g, 5 mmol, 0.25 eq.) was added drop-wise. The reaction mixture was stirred at room temperature for 14 h, another portion of catalyst was added (5 mmol) and stirring was continued for an additional 8 h. The solvent was removed and the crude mixture was separated on prep. LC (hexanes:ether:acetone, 4:1:1) to give 4.9 g (67%) of desired product as a viscous oil: 1 H NMR (300 MHz, CDCl3) Δ 8.1 (m, 2 H), 7.35 (m, 1 H), 4.95 (br s, 1 H), 4.6 (m, 1 H), 3.6-3.4 (m, 12 H), 3.4-3.1 (m, 5 H), 1.32 (d, J = 6 Hz, 3 H); MS (EI) 390.2 (M + Na).

3′, 4′, 5′-trimethoxy-2-methyl-6-(5-amino-indan-2-yloxy)-tetrahydro-pyran.
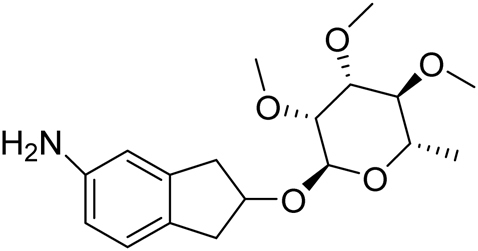


A solution of 3,4,5-trimethoxy-2-methyl-6-(5-nitro-indan-2-yloxy)-tetrahydropyran (3.4 g, 9 mmol, 1 eq.) in ethanol (100 mL) was placed in a Parr hydrogenation vessel, degassed by N_2_ bubbling, and 10% Pearlman’s catalyst (0.68 g) was then added. The bottle was pressurized with 40 psi H_2_ and shaken for 2 hr. The suspension was filtered through 2 filter papers, the solvent removed under reduced pressure to give 2.85 g (90%) of the product as a thick, tan oil: 1 H NMR (300 MHz, CDCl3) Δ 6.98 (d, J = 8 Hz, 1 H), 6.58 (d, J = 1 Hz, 1 H), 6.52 (dd, J = 8, 1 Hz, 1 H), 4.95 (br s, 1 H), 4.6 (m, 1 H), 3.6-3.4 (m, 12 H), 3.2-3.0 (m, 3 H), 2.88 (m, 2 H), 1.32 (d, J = 6 Hz, 3 H); MS (EI) 338.3 (M + Na).

Preparation of PBI based spinosyn mimic **4** - (7-Methyl-3-oxo-1,2,3,5-tetrahydro-benzo[4,5]imidazo[1,2-a]pyridine-4-carboxylic acid [2-((2 R,3 R,4 R,5 S,6 S)-3,4,5-trimethoxy-6-methyl-tetrahydro-pyran-2-yloxy)-indan-5-yl]-amide).
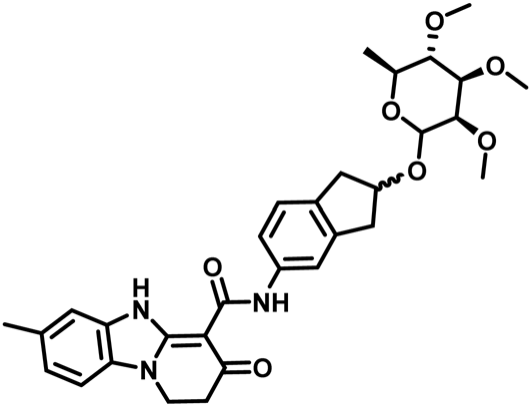


To a stirred solution of 600 mg (2.2 mmol) of the PBI ester and 900 mg (2.7 mmol) of the aniline (3,4,5-trimethoxy-2-methyl-6-(5-amino-indan-2-yloxy)-tetrahydro-pyran) in 10 mL of DCM under a nitrogen atmosphere was added 1.5 mL of 2 M solution of trimethylaluminum in DCM (3 mmol). The solution was allowed to stir for 3 h, then it was poured onto 10 mL of 1 N HCl and extracted with 20 mL of ethylacetate (EtOAc). The organic layer was dried and concentrated, then purified on a silica gel column, eluting with 10:10:10:10:1 hexanes:EtOAc:DCM:acetone:methanol to furnish 210 mg of PBI-SM as an off-white solid. 1 H NMR (300 mHz, CDCl3) δ 12.2 (br s, 1 H), 11.7 (br s, 1 H), 7.70 (s, 1 H), 7.35–7.05 (m, 6 H), 5.0 (s, 1 H), 4.62 (m, 1 H), 4.19 (t, J = 7 Hz, 2 H), 3.65-3.4 (m, 11 H), 3.25-3.10 (m, 3 H), 2.9 (m, 4 H), 2.45 (s, 3 H), 1.33 (d, J = 6 Hz, 3 H).

### Data and materials availability

Biological data sets generated for this paper are available from the corresponding author on reasonable request. The experimental compounds may be available in limited quantities from Dow AgroSciences under a materials transfer agreement.
